# In-Vivo Visualization of Tumor Microvessel Density and Response to Anti-Angiogenic Treatment by High Resolution MRI in Mice

**DOI:** 10.1371/journal.pone.0019592

**Published:** 2011-05-05

**Authors:** Roland T. Ullrich, Jan F. Jikeli, Michael Diedenhofen, Philipp Böhm-Sturm, Maike Unruh, Stefan Vollmar, Mathias Hoehn

**Affiliations:** 1 Max Planck Institute for Neurological Research, Cologne, Germany; 2 Center of Molecular Medicine, Cologne, Germany; 3 Department I of Internal Medicine, University Hospital, Cologne, Germany; Univesity of Texas Southwestern Medical Center at Dallas, United States of America

## Abstract

**Purpose:**

Inhibition of angiogenesis has shown clinical success in patients with cancer. Thus, imaging approaches that allow for the identification of angiogenic tumors and the detection of response to anti-angiogenic treatment are of high clinical relevance.

**Experimental Design:**

We established an in vivo magnetic resonance imaging (MRI) approach that allows us to simultaneously image tumor microvessel density and tumor vessel size in a NSCLC model in mice.

**Results:**

Using microvessel density imaging we demonstrated an increase in microvessel density within 8 days after tumor implantation, while tumor vessel size decreased indicating a switch from macro- to microvessels during tumor growth. Moreover, we could monitor in vivo inhibition of angiogenesis induced by the angiogenesis inhibitor PTK787, resulting in a decrease of microvessel density and a slight increase in tumor vessel size.

**Conclusions:**

We present an in vivo imaging approach that allows us to monitor both tumor microvessel density and tumor vessel size in the tumor. Moreover, this approach enables us to assess, early-on, treatment effects on tumor microvessel density as well as on tumor vessel size. Thus, this imaging-based strategy of validating anti-angiogenic treatment effects has high potential in applications to preclinical and clinical trials.

## Introduction

In the past years preclinical and clinical studies have demonstrated the essential role of angiogenesis for initiation of tumor growth [1], [2]. Treatment strategies inhibiting angiogenic processes mainly targeting the vascular endothelial growth factor (VEGF) and its receptor (VEGFR) have been implicated in clinical trials. Thus, non-invasive methods to visualize and to monitor tumor angiogenesis, and its inhibition, respectively, are of high clinical relevance.

Currently, dynamic contrast enhanced magnetic resonance imaging (DCE-MRI) is in clinical use for the assessment of anti-angiogenic treatment effects [3], [4]. DCE-MRI represents an indirect measure of angiogenesis since it mainly reflects leakage of the vascular bed by measuring the transfer of contrast agent into the interstitial space. Due to high VEGF levels within tumors vascular leakage is increased in tumor microvessels. Thus, DCE imaging is proposed to be an accurate marker to detect therapeutic VEGF inhibition. Gadolinium-based contrast agents are mostly used for DCE-MRI.

Dennie et al proposed the use of the ratio of gradient echo and spin echo relaxation rate changes (ΔR*_2_/ΔR_2_) after injection of a high molecular weight contrast agent to measure average microvessel density within a voxel [5]. These authors found a good correlation between the MRI derived in vivo data and histology. Based on these findings Jensen and Chandra proposed to map the ratio of Q = ΔR_2_/(ΔR*_2_)^2/3^ and demonstrated that Q is dependent on water diffusion but independent of the concentration of the contrast agent [6]. Because of the heterogeneity of diffusion within tumors and changes of diffusion during tumor growth [7] we sought to establish a multi-echo spin echo sequence that takes the tumor diffusion into account for the determination of tumor microvessel density and tumor vessel size.

In this study, we present an in vivo MRI approach that allows for simultaneous assessment of tumor microvessel density and vessel size by the use of a superparamagnetic iron oxyde (SPIO) at a very high spatial resolution. We validated the accuracy of this approach by monitoring tumor angiogenesis and detecting response to the VEGFR/PDGFR tyrosine kinase inhibitor vatalanib in a NSCLC xenograft model in mice.

## Materials and Methods

### Cell Culture

We used the NSCLC cell line H1975 [8]. Cells were maintained in RPMI 1640 supplemented with 10% heat inactivated fetal bovine serum (FBS, Roche Diagnostics, Mannheim, Germany), 1% penicillin and 1% streptomycin (P/S, Life Technologies) at 37°C in a 5% CO2/95% air atmosphere.

### Xenograft model

All animal procedures were in accordance with the German Laws for Animal Protection and were approved by the local animal committee and the local authorities (LANUV, Recklinghausen, reference number: 8.87-50.10.31.08.331).

Tumors were generated by s. c. injecting 5×10^6^ H1975 tumor cells into *nu/nu* athymic male mice as described recently [8] (Janvier, Europe). In the first set, we longitudinally measured animals on days 1 (n = 4), 4 (n = 6, 2 sacrificed for immunohistochemistry), 8 (n = 6, 2 sacrificed for immunohistochemistry), 14 (n = 6, 2 sacrificed for immunohistochemistry), 21 (n = 6, 2 sacrificed for immunohistochemistry) after tumor cell injections. In the second set, animals were randomized into two groups, vehicle treated (control) and vatalanalib (PTK787) treated. Vehicle and PTK787 animals were studied on day 8 (start of treatment) 14, and 21 after tumor cell implantation (vehicle n = 6, PTK787 n = 6; 2 sacrificed for immunohistochemistry on day 21). Mice were treated daily by oral gavage of 75 mg/kg PTK787. PTK787 was dissolved at 1% DMSO and 0.5% Tween 80 in distilled water. All controls were dosed with the same volume of vehicle (1% DMSO and 0.5% Tween 80).

### Immunohistochemistry

After the last MRI measurements, animals were sacrificed and s.c. tumors were extracted. Tumors were embedded in tissue freezing medium (Jung, Nussloch, Germany) and cut in 10-µm frozen sections. Hematoxylin Eosin staining on the tissue was performed according to standard protocols. Microvessel density was assessed with CD31 staining (1∶50 dilution, Mat.-No. 550274, BD Pharmingen™). CD31 positive endothelial cells or cell cluster were counted. In order to determine the mean number of microvessel density within the tumor, the number of CD31 positive cells was determined by 3 different areas with maximal, moderate, and minimal endothelial density. The mean number of microvessels was determined as (F1+F2+F3)/3.

### Magnetic Resonance Imaging (MRI)

All experiments were performed on an experimental animal scanner at 7T (Bruker BioSpec; Bruker) equipped with a gradient set of 400 mT/m. Radio frequency (RF) irradiation and signal detection was achieved with custom-built coils: a 8-cm-diameter Helmholtz coil arrangement for RF excitation, and a 16 mm diameter surface coil for signal detection.

To determine ΔR2 and ΔR2* maps multi slice multi spin echo (MSME) and multi gradient echo (MGE) pulse sequences, respectively, were performed before and after injection of iron oxide nanoparticles (Endorem®, Guerbet Inc.) at a dose of 30 mg Fe/kg. The postcontrast image acquisition was delayed by 2 min to ensure a steady-state distribution of contrast agent in the vascular network.

MSME and MGE MR images were obtained with the same Field of View (FOV) (16 mm×16 mm), and matrix size (64×64), and a slice thickness of 0.3 mm (Matrix size 250×250×300 µm^2^).

MSME was acquired with TR = 5000 ms and TE = (10.9, 21.8,…,109)ms. MGE was acquired with TR = 1400 ms and TE = (4, 8,…,32) ms with a 60° hermite pulse.

To map the apparent diffusion coefficient (ADC) of water, two diffusion-weighted images with b = 300, b = 800 s/mm^2^, in both, x and z directions of the gradient system were acquired, together with a reference image (b≈0 s/mm^2^) at the following parameters: voxel size = 0.5×0.25×0.3 mm^3^, zero-filled to 0.25×0.25×0.3 mm^3^, matrix 64×64. The total scan time was 49 min and 16 seconds. We started the measurement with the ADC map. Then, we acquired the MSME and MGE pre CA-injection datasets. The post injection scan time was 16 min and 38 sec. The signal intensity after contrast agent injection was stable within half an hour.

### Data analysis

The co-registration of the images was performed using FSL software (FLIRT, Oxford, UK). IDL was used for image processing (Interactive data language, ITT, VIS). We used a volume of interest (VOI) of the entire tumor to assess the global values of the ADC, MDI and VSI. The in-house developed software VINCI was used for volume of interest (VOI) analysis of MR images [9].

The ADC map was calculated from the diffusion-weighted images with a mono-exponential fit of the signal intensity of the three different b values (b_0_, 300, 800 s/mm^2^).

The ΔR*_2_ maps were determined with the second echo (TE = 8 ms) by
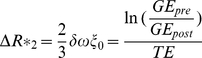
(1)


[10], [11] and a modified calculation for multi-spin echo sequences with the third echo (TE = 32.7 ms)
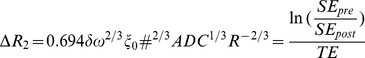
(2)


[12] (ξ_0_  = Blood Volume fraction; δω = 2πγΔ*Χ*B_0_ = frequency shift, # = number of 180° pulses; R = Vessel Size Index (VSI) Δ*Χ* = changes in the susceptibility, B_0_ = magnitude of the magnetic field, γ = gyromagnetic ratio). The prefactor 0.694 had been calculated in [12].

Based on equation (2) we calculated the microvessel density index (MDI) from 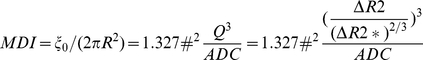
(3)


**Figure 1:**
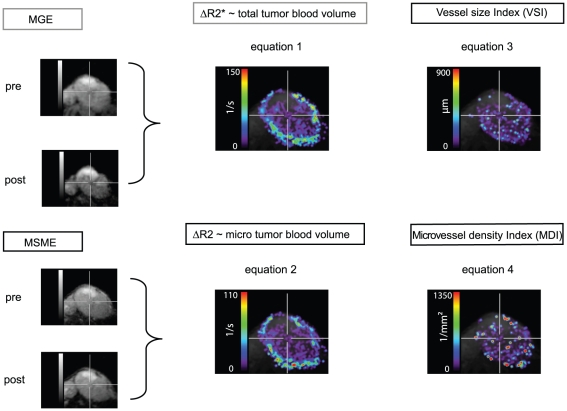
Schematic of data calculation for the microvessel density index (MDI) and the vessel size index (VSI).

As such, measurement values are independent of the local contrast agent concentration. The process is schematically depicted in Fig. 1.

The combination of equation (1) and (2) leads to the term of the VSI 
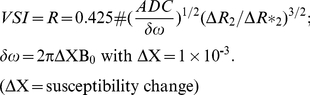
(4)


[13].

### Statistical analysis

Statistical tests were performed using SPSS software (release 18.0 SPSS, Inc., Chicago. IL.USA). To assess statistical significance we used the Spearman correlation and the t-test. Statistical significance was set at *p*<0.05. Values are indicated as mean and standard deviations.

## Results

### Model

Equation (2) considers the number of 180°-pulses (#) within the MSME sequence. The calculation of the VSI map and the MDI map are based on equation (2). To our knowledge, we take for the first time the number of 180°-pulses into account by calculating VSI and MDI maps.

### Comparison of the image derived MDI values with histology

**Figure 2:**
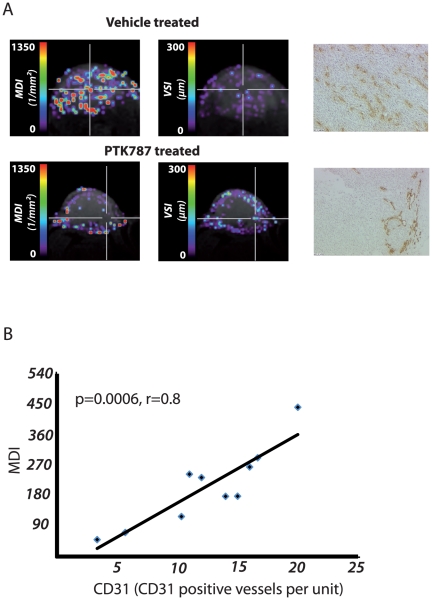
Comparison of the image derived values of MDI and VSI to CD31 positive endothelial cell staining. (A) a vehicle treated (upper row) tumor presenting a high microvessel density index (MDI (1/mm^2^)) in MRI and a low vessel index (VSI (µm)). The PTK787-treated tumor (lower row) presents low MDI values with high VSI values. (B) correlation between CD31 positive vessels per area unit and the imaged derived MDI values (n = 10).

In order to validate our image-derived data for microvessel density we compared the MDI values to the in vitro microvessel density. The in vitro microvessel density index was immunohistochemically assessed by CD31 positive microvessels at different time points directly after the MRI measurements. Here, we found a significant correlation between the in vivo derived MDI values and the microvessel density index of CD31 positive cells (r = 0.8, p = 0.0006; *Spearman correlation*) (Fig. 2). Furthermore, tumor areas with high VSI values showed also large vessel size in histology (Fig. 2A).

### Monitoring tumor microvessel density and vessel size

**Figure 3:**
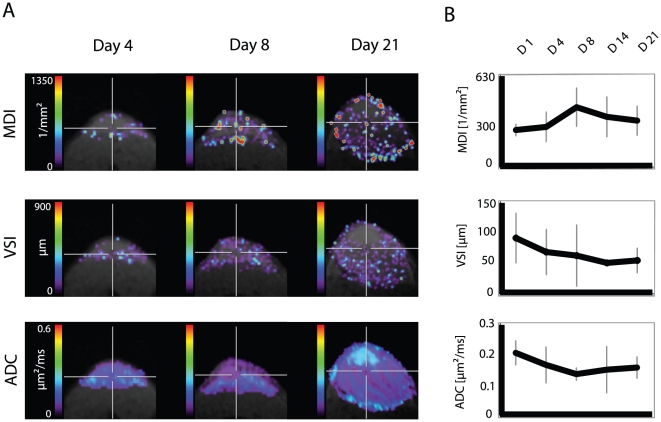
Longitudinal investigation of vascular dynamics during tumor growth. Simultaneous in vivo monitoring of tumor depiction with T2-weighted imaging, microvessel density (MDI), vessel size (VSI) and apparent diffusion coefficient (ADC) during tumor growth on day 1, day 4, day 8, day 14, and day 21 (D1, D4, D8, D14, D21).

We analyzed the growth of tumor microvessels as detected by the MDI map from day 1 to day 4, 8, 14, and 21. Already one day following s.c. inocculation of the tumor xenograft we observed high intra- and subcutaneous MDI values around the tumor. On day 4 we found an increasing number of tumor microvessels within the tumor (MDI value: 198±28). The maximum was reached 8 days after tumor cell inocculation (MDI value: 297±73). The MDI values then slightly decreased at day 14 (247±94) and day 21 (230±72). We did not find significant changes between day 1 and 4, day 4 and 8, day 8 and 14, day 14 and 2. Of note, at later time points we found a rather homogeneous distribution of the microvessels within the center as well as in the outer rim of the tumor (Fig. 3). This was accompanied with a homogeneous, rather low ADC value on days 14 and 21 (Fig. 3).

Using maps of the vessel size index (VSI) we estimated the vessel diameter over time. Interestingly, we found inverse behavior of the vessel size and vessel density changes: an increase in MDI from day 4 to day 14 was accompanied by a decrease in VSI, indicating a switch from larger to smaller vessel sizes (VSI, day 4: 68.7±40.8; day 14: 50.7±1.8). Tumor vessels with larger diameter were found in the outer rim of the tumor with their diameter still increasing during the following days (Fig. 3).

ADC values showed a decrease within the first 8 days reflecting a decline in extracellular diffusion, most probably due to an increase in tumor cell density.

### Response to PTK787 treatment

**Figure 4:**
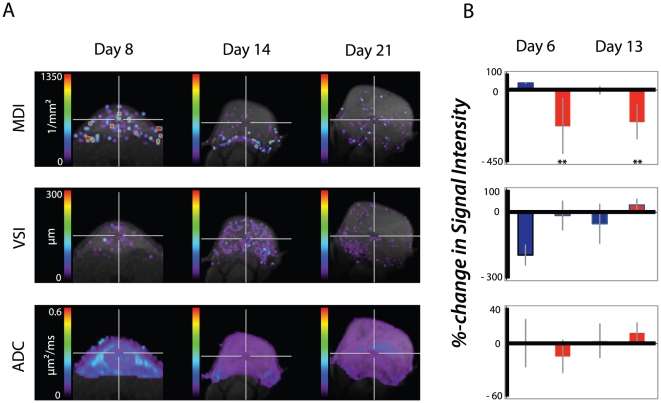
Effect of treatment on tumor angiogenesis. (A) Monitoring PTK787 treatment effects on changes in tumor microvessel density index (MDI), tumor vessel size (VSI) and apparent diffusion coefficient (ADC) during tumor growth and treatment. PTK787 treatment started at day 8 after tumor inoculation. (B) Quantitative analysis of vascular variables after 6 days and 13 days of PTK787 treatment. The blue bars represent the vehicle treated tumors and the red bars the PTK787 treated tumors.

We determined the potential of our protocol to assess response to anti-angiogenic treatment. Tumors were grown for 8 days and MDI, ADC and VSI were measured. We then started PTK787 treatment and monitored response to treatment after 6 and 13 days by calculating percentage changes in MDI, VSI and ADC. Already after 6 days of oral PTK787 treatment, we found a significant decline in the MDI values in comparison to the vehicle group (p = 0.021) (see Table 1 and Fig. 4). This was confirmed after 13 days of treatment (p = 0.005). In contrast, the VSI decreased in the control group over time whereas in the treated tumors the VSI remained nearly stable at day 6 and even increased till day 13. This indicates a switch from macro- to micro-vessels in the vehicle group. On the other hand, PTK787 treatment induces the inverse effect resulting in a shift from micro- to macrovessels.

**Table 1:** Percent changes (relative to day 0) in T2, MDI, VSI and ADC values after 6 days and 13 days of PTK787/vehicle treatment.

	*PTK787 treated*				*Vehicle treated*			
	day 6		day 13		day 6		day 13	
	Mean	SD	mean	SD	mean	SD	mean	SD
**T2**	−4.79	3.85	−0.93	16.85	−5.69	5.08	−22.55	16.85
**MDI**	−225.10	169.88	−199.52	104.80	41.30	2.17	−3.82	23.22
**VSI**	−15.85	64.90	31.63	26.60	−194.20	45.30	−53.10	87.90
**ADC**	−14.61	18.32	11.71	11.45	0.22	26.72	2.84	18.97

In parallel we observed a decrease in the ADC value after 6 days of PTK787 treatment whereas there was nearly no change in the ADC in the vehicle treated tumors. The ADC value then increased till day 13 during PTK787 treatment (Tab. 1 and Fig. 4).

## Discussion

In this study we demonstrate the feasibility to non-invasively determine microvessel density in an experimental NSCLC model by the use of the MRI derived MDI map. Moreover, we propose a protocol permitting simultaneous monitoring of tumor vessel size (VSI) and tumor microvessel density (MDI). We show that the values obtained by the MDI map reflect the microvessel density as assessed by immunohistochemical CD31 staining. Most importantly, the MDI allows to monitor PTK787 induced reduction in microvessel density as early as 6 days after start of treatment. Finally, repetitive MR imaging reveals a shift of vessel diameter toward larger lumen in PTK787 treated tumors in vivo.

Currently, there are numerous anti-angiogenic agents in clinical trials ([14]; http://www.cancer.gov/clinicaltrials). Likewise, new imaging modalities are required that allow to monitor tumor microvessel density growth and the detection of anti-angiogenic treatment effects. As reported previously, MR imaging offers the detection of capillaries with a diameter of 10 to 30 µm, reflecting the range of the diameter of tumor microvessels [6], [10]. Jensen [15] first proposed the Q-map for the assessment of microvessel density in vivo. Based on this approach, Wu et al found reasonable Q-map values within the healthy mouse brain when comparing the results with histological analysis [16]. However, as indicated by Jensen in 2006 [15], the diffusion within the tissue affects the calculation of microvessel density (cf. Eq. 3). This is particularly true for tumor tissue since the diffusion within the tumor is highly variable. Therefore, we established a new protocol for vessel density imaging that includes the ADC map, to take the function of the diffusion heterogeneity within the tumor into consideration for the determination of the MDI. Herewith, we received a clear improvement of the vessel density values, as demonstrated with the significant correlation to the microvessel density assessed by immunohistochemistry. Of note, the same calculation without the ADC map showed no correlation to immunohistochemically assessed by microvessel density (data not shown).

The theoretical description of Kiselev et al [11] has been made for a single echo experiment. Nevertheless, multi echo experiments were used to calculate the Q-maps or the VSI [17]. The gradients for the 180 degree pulses induce diffusion weighting. By using multi spin echo sequences this diffusion weighting is usually not considered. Therefore, the calculation of delta R2 via the fitting on multi echo sequences leads to incorrect results for the VSI and MDI. Thus, we applied the correction factor as suggested by Kiselev [12] to take the diffusion weighting into account. As the correction factor is considered in the formula (3) and (4) both the second and the third echo can be used. However, by using further 180°-pulses we found an increase of the obtained MDI that did not reflect the results from the immunohistochemistry. Thus, we conclude that it is important to use a spin echo sequences with the appropriate echo time to calculate the MDI and VSI.

We have presented a new strategy for the simultaneous estimation of the vessel size index (VSI) and the microvessel density index (MDI) by the use of a multi spin echo sequence. With this tool, we could demonstrate in vivo that in untreated tumors the decrease in the mean tumor vessel size is paralleled by an increase in microvessel density. These findings are in line with a study by Drevs et al. [18] who observed a shift of vessel diameter toward larger lumen in PTK787 treated tumors in comparison to the vehicle group in vitro. This observation is reasonable since it reflects the sprouting of smaller tumor microvessels from pre-existing larger vessels during tumor growth that is inhibited by PTK787.

After 6 days of PTK787 treatment, we found a decrease in the ADC value. It is well known that inhibition of VEGF/VEGFR2 results in a reduction in vascular permeability [19]. We hypothesize that the PTK787 induced decline in vessel permeability reduces interstitial edema and, thus, intra-tumoral diffusion that is reflected by the observed decrease in ADC map. Moreover, after 13 days of treatment there was again a slight increase in the ADC value most probably due to necrotic tumor transformation [20], [21], [22], [23].

Finally, we have demonstrated that the MDI method permits the characterization of microvessel density in vivo in longitudinal studies. Further, the MDI detected the PTK787 treatment induced reduction of microvessel density as early as 6 days of treatment. This is of high clinical interest since it allows for monitoring effects of anti-angiogenic treatments based on the growth and sprouting of tumor microvessels. Of note, the iron oxide nanoparticle Endorem^R^ is a true intravascular contrast agent with a long plasma half-life (T1/2>2.5 h) [24], already approved for human use. Severe side effects have been reported for the gadolinium based contrast agents [25]. Thus, Endorem represents a highly promising contrast agent for clinical studies. Moreover, using Gd-complexes the changes of the signal induced by the susceptibility is weak. Since Endorem is a paramagnetic CA the changes of the local susceptibility in the steady state is much higher than induced by gadolinium complexes.

In summary, we present an in vivo imaging approach for simultaneous monitoring of tumor microvessel density (MDI) and tumor vessel size (VSI). This approach enables the early assessment of treatment effects on microvessel density as well as on tumor vessel size. Thus, this imaging method bears high potential for monitoring anti-angiogenic treatment effects in preclinical and clinical trials.
